# Health as an independent predictor of the 2017 French presidential voting behaviour: a cross-sectional analysis

**DOI:** 10.1186/s12889-019-7861-3

**Published:** 2019-11-06

**Authors:** Jean-David Zeitoun, Matthieu Faron, Sophie de Vaugrigneuse, Jérémie H. Lefèvre

**Affiliations:** 1 Centre d’Epidémiologie Clinique, Hôtel Dieu Hospital, Assistance Publique-Hôpitaux de Paris, Paris, France; 2 Department of Gastroenterology and Nutrition, Hôpital Saint-Antoine, Assistance Publique-Hôpitaux de Paris, Paris, France; 3 Department of Proctology, Groupe Hospitalier Diaconesses-Croix Saint-Simon, Paris, France; 4 Departments of Surgical Oncology and Department of Biostatistics and Epidemiology, Gustave Roussy Cancer Campus Grand Paris, Villejuif, France; 5 INSERM U1018 CESP, Université Paris-Sud, Université Paris-Saclay, Villejuif, France; 6 Sciences Po, Paris, France; 7 Department of Digestive Surgery, AP-HP, Hôpital Saint Antoine, Sorbonne Université, F-75012 Paris, France; 8 INSERM, UMRS 938 - Centre de Recherche Saint-Antoine, Equipe “Instabilité des Microsatellites et Cancers”, Equipe labellisée par la Ligue Nationale contre le Cancer, F-75012 Paris, France

**Keywords:** Health status, Socioeconomic indicators, Voting pattern

## Abstract

**Background:**

It has been suggested that poor health has influenced vote for Brexit and the US presidential election. No such research has been published regarding the 2017 French presidential election.

**Methods:**

We performed a cross-sectional analysis using a comprehensive set of socioeconomic and health indicators, to be compared with voting outcome at the first round of the 2017 French presidential election. The 95 French departments were selected as the unit of analysis. Data were obtained from publicly available sources. The linear model was used for both univariate and multivariate analysis to investigate the relation between voting patterns and predictors. Sensitivity analyses were done using the elastic-net regularisation.

**Results:**

Emmanuel Macron and Marine Le Pen arrived ahead. When projected on the first factorial plane (~ 60% of the total inertia), Emmanuel Macron and Marine Le Pen tended to be in opposite directions regarding both socioeconomic and health factors. In the respective multivariate analyses of the two candidates, both socio-economic and health variables were significantly associated with voting patterns, with wealthier and healthier departments more likely to vote for Emmanuel Macron, and opposite departments more likely to vote for Marine Le Pen. Mortality (*p* = 0.03), severe chronic conditions (*p* = 0.014), and diabetes mellitus (*p* < 0.0001) were among the strongest predictors of voting pattern for Marine Le Pen. Sensitivity analyses did not substantially change those findings.

**Conclusions:**

We found that areas associated with poorer health status were significantly more likely to vote for the far-right candidate at the French presidential election, even after adjustment on socioeconomic criteria.

## Introduction

Two great democracies have recently encountered outcomes that were considered as unexpected and that have been interpreted as reflecting a rejection of established political parties. Namely, vote for Brexit in United-Kingdom (UK) and Donald Trump’s election had not been anticipated by most polling institutes [1, 2]. Even though multiple explanatory factors were thought to be involved for each, it has been argued that health and healthcare had a significant influence on voters [3]. In the UK, self-reported health has been measured as declining since 2010 and death rate in most age categories rose over the last years [4]. Moreover, public healthcare funding was a key issue in political debates, with proponents pretending that Brexit would bring substantial savings for the UK that would be subsequently reallocated toward the National Health System, a claim that has been rapidly retracted just after the vote [5]. In the US, it has been shown that the strongest factor associated with an increase in Republican voting for Donald Trump, as compared to prior elections, was a decrease in health status when measured by several indicators [6]. In the US also, the healthcare system and its funding were paramount in the campaign debates although Donald Trump’s intentions to repeal Affordable Care Act have not succeeded yet [7]. Even though those elements cannot be considered as definitive evidence, they strongly suggest that health and healthcare topics have been indeed important factors of citizen choices in the UK and US votes. This would be consistent with prior peer-reviewed work showing that health may influence attitude toward democracy and turnout to elections [8, 9]. However, in-depth studies regarding true votes and psychological factors that could have driven behaviours are lacking, despite rapidly accumulated evidence that those two western countries may be failing to maintain life expectancy [10, 11].

**Fig. 1:**
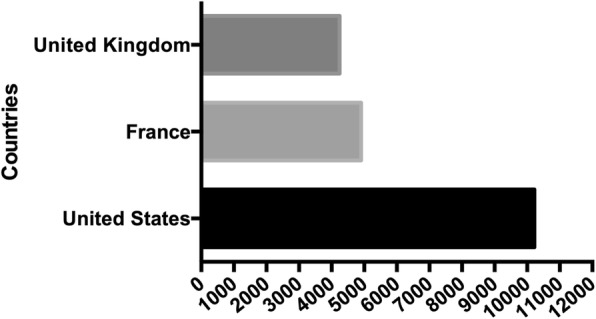
Recent health expenditures of the United-Kingdom, the United States (US) and France according to the Organisation for Economic Cooperation and Development (US dollars, per capita)

France also has issues with people’s health and its healthcare system. According to official estimates, there has been an unexpected peak of deaths in 2015, with no clear explanation [12, 13]. Even though France’s healthcare system is frequently considered as equitable, budgets are highly pressured and concerns over sustainability of the current model are growing [12, 14]. For the sake of comparison between the three countries, a graph has been constructed, showing health expenditures over the last years of the UK, the US, and France, according to the data established by the Organisation for Economic Cooperation and Development (Fig. 1). Soon after the UK and US votes, France entered into a major political campaign, namely the 2017 presidential election. After the first round, two candidates were selected for a second round in accordance with the established rules of the country’s 5th Republic. Emmanuel Macron (EM), a novel politician claiming a “nor left-nor right wing” position and Marine Le Pen (MLP), the far-right leader competed for the second and final round, as foreseen by most polling institutes. Even though Emmanuel Macron won by far the election, Marine Le Pen’s votes both at the first and second round reached unprecedented levels for a far-right party in France, with about 11 million people voting for her at the second round. While social factors, like unemployment, are strongly linked with turnout and voting behaviour [15], we are unaware of any investigation regarding possible relationships between health and voting patterns in France whereas, like in many high-income democracies, our healthcare system experiences difficulties, both regarding funding and inequalities [12]. Moreover, the presidential campaign has emphasized people’s concerns regarding funding of the combined set of public and private health insurances that characterizes the French healthcare system [14]. Since the 2017 presidential election, health and economic issues have been placed at the top of the political agenda [16] but we still lack data regarding a possible association between health status and voting patterns. Such measures would help to determine whether recent trends observed in the UK and the US reflect a more general phenomenon, to address concerns of people living in areas affected by poorer health indicators, and to inform the overall public health debate.

Therefore, we sought to analyse possible associations between voting patterns at the first round of the 2017 French presidential election and relevant health indicators. Since there is a well-known relationship between wealth and health, [17, 18] we aimed to adjust our analyses on several socioeconomic indicators.

## Methods

### Data sources

We used official and publicly available databases to retrieve and gather the data we needed for the study: the National Institute for Statistics and Economic Studies (INSEE, *Institut National de la Statistique et des Etudes Economiques*), the Directorate of Research, Evaluation, and Statistics (DREES, *Direction de la Recherche, des Etudes, des Evaluations et des Statistiques*), the French Public Health Insurance (*Assurance Maladie*), the Health Watch Institute (*Institut de Veille Sanitaire*, now merged into another structure called *Santé Publique France*), the Observatory of Inequalities (*Observatoire des Inégalités*), and the Research Institute in Health Economics (IRDES, *Institut de Recherche et Documentation en Economie de la Santé*). In each data source, the relevant data were collected for the most recent year available, and classified according to the geographic area of analysis, namely the so-called 95 French departments. Overseas departments and territories were not included in the current study for the sake of analysis.

### Health indicators

For each French department, we retrieved the following health indicators (year of availability between brackets): mortality (2015), life expectancy (2015), prevalence of people affected by at least one significant chronic condition, as recognized and entirely covered by the French Public Health Insurance (2014), prevalence of diabetes mellitus (2013), asthma (2015) and chronic obstructive pulmonary disease (COPD) (2014). Those indicators were chosen because they were both thought to be relevant and because publicly available information is known to be highly reliable and accurate. Some of those indicators have already been used by other authors [19, 20], whereas others like asthma and COPD are more original. However, we believe that the latter are also important because they represent frequent and significant chronic conditions.

### Wealth and other social indicators

Similarly, the following variables were collected and categorized according to the geographic unit of analysis: rate of people under the poverty line (2010), rate of people covered by the main public unemployment allowance (so-called Solidarity Labour Income, *Revenu de Solidarité Active*) (2015), rate of unemployment (2016), median wages (2010), level of inequalities as measured by the Gini Coefficient [21] (2004), and rate of single parental families (2013). Those variables were again chosen for their relevancy and because they are rigorously maintained by the French government. Many of them reflect indicators already chosen by other authors in prior recognized research [22–25].

### Voting patterns

Data regarding voting rates and results in each French department at the first round of the presidential election were retrieved from the publicly available database maintained by the Ministry of Internal Affairs, which displays the official results of the presidential election just the day after the vote. Of note is that vote is not mandatory, and there is no reward or fine for participation or lack thereof.

### Statistical analysis

Quantitative data are represented as means (± Standard Deviation). All frequency variables are represented as percentage. The univariate relation between an independent variable and the probability to vote for one candidate was estimated by a single variable linear regression model. Nonlinear relationship between the dependent and independent variables were initially investigated by introducing a quadratic term in the formula. However, graphical exploration of the relation showed that variables with significant quadratic terms were often highly influenced by outliers inducing a risk of over fitting the model. Thus the “simple” and parsimonious linear relation was used for all the variables. Any variable achieving a *p* < 0.1 significance in the univariate analysis was considered for the multivariate model using multiple linear regression. A backward stepwise selection procedure based on the Akaike Information Criteria was used to select the best subset of variables. French departments are heterogeneous in terms of population size, ranging from 76,607 to 2,595,536 people. Therefore, all linear models were weighted by population size. Sensitivity analyses were done to test the robustness of the results: first regressions were made without weighting by population size to test the impact of this parameter. Secondly, elastic-net regression (which is a more robust method in presence of highly correlated covariates and when the number of predictors is high in comparison with the number of observations) was used to confirm the variable selection.

Principal component analysis (PCA) was based on all health, wealth and social indicators after scaling and weighting by the departments’ population. Voting patterns were not used to construct the axis but were subsequently projected on the factorial plane. PCA is a common statistical procedure used in data exploration in presence of multiple numerical predictors. The total variability in the dataset is reduced to two (or more) principal components which are like “new variables” summarizing the older ones. The correlations between each variable and the principal component are calculated and allow seeing which variables go together. Supplementary variables, which were not used to construct the principal components, can be represented on the factorial plane to see whether or not they go together and how they relate to the variables.

All tests were bilateral and *p*-value< 0.05 was used to denote statistical significance. All analyses were done with the R 3.4.0 software (the R Core team, Vienna Austria) and noticeably, the packages ggplot2 2.2.1 (Wickham 2009), FactoMineR 1.35 (Le, 2008) and glmnet (Friedman, 2010) 2.0–10.

## Results

**Fig. 2:**
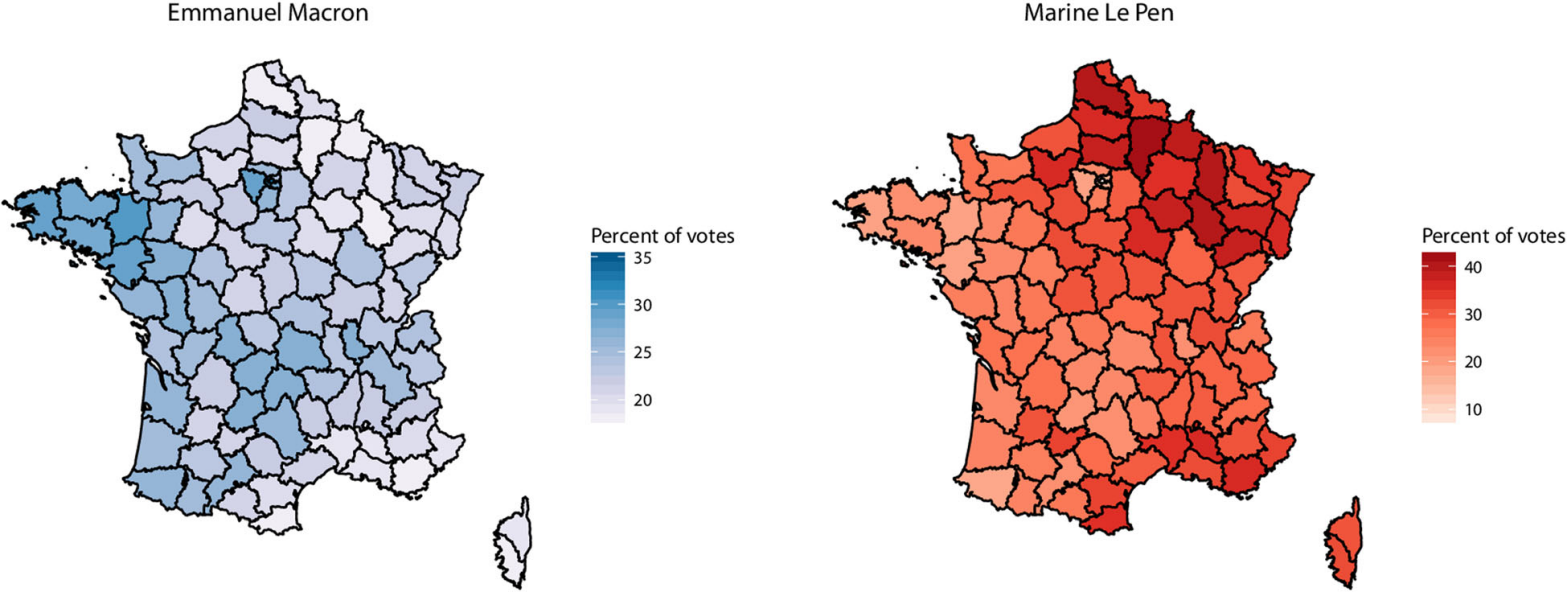
Results of the first round of the 2017 French presidential election for the two leading candidates, Emmanuel Macron and Marine Le Pen in the 95 French departments. Administrative boundaries shapefiles were obtained from the Global Administrative Boundaries project (GADM) from UC Davis (https://gadm.org/index.html). Their data are freely available for academic use and creating maps for academic publishing is allowed. Choropleth map were then generated with the R 3.15 software (The R Core Team, Vienna Austria) and the ggplot2 package (Wickham, 2018, https://ggplot2.tidyverse.org) both open sources software

The 95 departments were included in the study, with a median population of 536,694 per department [76,607-2,595,536]. Departments’ characteristics with respect to socioeconomic and health indicators are presented in Table 1. The outcome of the first round of the election for both leading candidates is represented on the French map in Fig. 2. Abstention rate was 22.23%.

**Table 1:** Characteristics of the 95 French departments

	Mean (± SD)
Median annual wages (k€)	19.603 (± 2.272)
People under the poverty line (%)	14.5 (± 2.9)
Unemployment (%)	9.9 (± 1.8)
Solidarity Labour Income (%)	3.4 (± 1.0)
Inequality (Gini coefficient)	3.0 (± 0.3)
Single parent family (%)	20.7 (± 2.9)
Life expectancy (years)	81.93 (±0.89)
Mortality (%)	1.0 (± 0. 2)
Chronic condition (%)	18.9 (± 2.2)
COPD (%)	2.9 (± 0. 4)
Asthma (%)	8.9 (± 1.9)
Diabetes mellitus (%)	4.7(± 0.5)

Data are given as mean of percentage unless otherwise stated. COPD stands for Chronic Obstructive Pulmonary Disease

### Principal component analysis

**Fig. 3:**
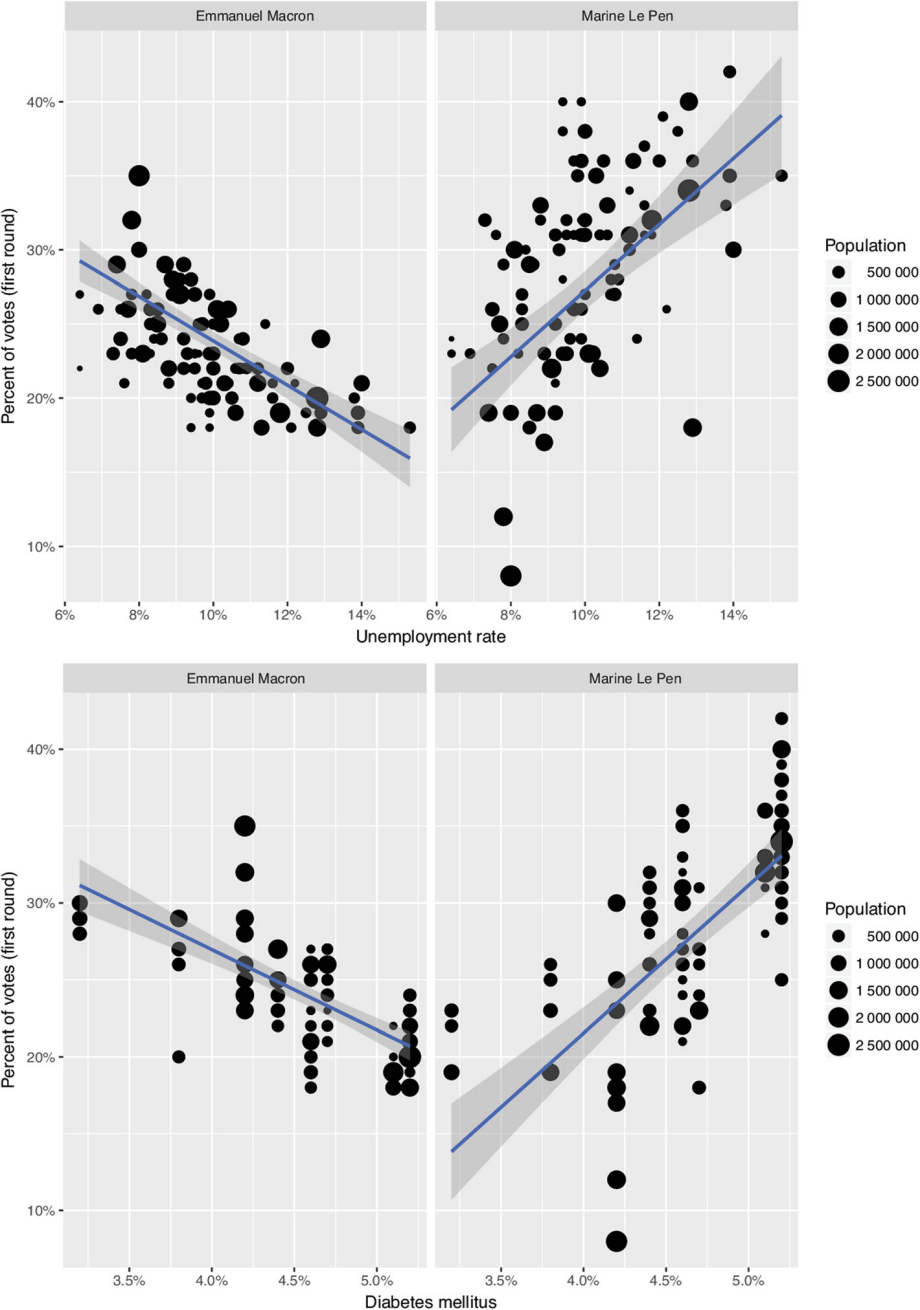
Linear regressions of impact of unemployment (top) or diabetes mellitus (bottom) on vote for Emmanuel Macron (left) and Marine Le Pen (right) among the 95 French departments. Each point represents a department, with its size being commensurate to its population. The blue line is the regression line and the shaded part its 95% confidence interval. EM: Emmanuel Macron, MLP: Marine Le Pen

The first and second principal components (PC) explained the majority of the total variability with respective inertia of 33.7 and 26.2%. The variables which contributed to more than 10% of the first PC were unemployment, poverty, solidarity labour incomes and life expectancy. Those for the second PC were inequalities, median wages, single parental family, asthma and mortality. Figure 3 shows the first factorial plane. In this plane, EM votes and MLP votes were nearly in opposite directions, in particular in the first PC axes.

### Univariate analysis

In the univariate analysis, all variables were significantly associated with voting pattern for EM and MLP, except for single parent’s family (*p* = 0.42, and *p* = 0.23 respectively) (Table 2 and Table 3). As illustrated for diabetes mellitus and unemployment in Fig. 3, associations between the voting patterns and the variables were in opposite directions, that is negative coefficient for EM while coefficient for MLP was positive and vice versa, except for single parent family (see Table 2 and Table 3).

**Table 2:** Univariate and multivariate analysis of predictors of vote in favour of Emmanuel Macron based on 95 French departments

	Univariate		Multivariate	
	Coefficient (±SD)	p*	Coefficient (±SD)	p*
Median annual wages(k€)	0.009 (±0.001)	< 0.0001	0.0036 (±0.002)	0.048
Poverty	−0.613 (±0.111)	< 0.0001		
Unemployment	−1.496 (±0.171)	< 0.0001	−0.71 (±0.15)	< 0.0001
Solidarity Labour Income^$^	−1.671 (±0.346)	< 0.0001		
Inequality	4.119 (±0.776)	< 0.0001	2.44 (± 0.795)	0.003
Single parent family	−0.112 (±0.14)	0.42		
Life expectancy (years)	0.024 (±0.003)	< 0.0001		
Mortality	−8.872 (±1.898)	< 0.0001	2.33 (±1.37)	0.09
Chronic condition	−0.966 (±0.167)	< 0.0001	−0.272 (±0.106)	0.011
COPD	−3.526 (±0.904)	0.00018		
Asthma	0.49 (±0.167)			
Diabetes mellitus	−5.231 (±0.576)	< 0.0001	−3.33 (±0.40)	< 0.0001

COPD: Chronic Obstructive Pulmonary Disease. Coefficients are coefficients of linear regression. * Linear regressions were weighted by department population

^$^ While significant in the univariate analysis, not included in the multivariate due to collinearity with unemployment

**Table 3:** Univariate and multivariate analysis of predictors of vote in favour of Marine Le Pen based on 95 French departments

	Univariate		Multivariate	
	Coefficient (±SD)	p*	Coefficient (±SD)	p*
Median annual wages(k€)	−0.015 (±0.002)	< 0.0001		
Poverty	0.744 (±0.223)	0.0012		
Unemployment	2.232 (±0.356)	< 0.0001	0.76 (±0.26)	< 0.0001
Solidarity Labour Income^$^	2.068 (±0.678)	0.003		
Inequality	−8.434 (±1.375)	< 0.0001	−6.90 (±0.94)	< 0.0001
Single parent family	−0.313 (±0.256)	0.23		
Life expectancy (years)	−0.048 (±0.005)	< 0.0001		
Mortality	17.22 (±3.444)	< 0.0001		
Chronic condition	1.513 (±0.322)	< 0.0001	0.32 (±0.21)	0.12
COPD	8.877 (±1.541)	< 0.0001	3.25 (±1.11)	0.004
Asthma	−1.132 (±0.299)	0.00027		
Diabetes mellitus	9.626 (±1.058)	< 0.0001	6.57 (±0.80)	< 0.0001

COPD: Chronic Obstructive Pulmonary Disease. Coefficients are coefficients of linear regression. * Linear regressions were weighted by department population

^$^ While significant in the univariate analysis, not included in the multivariate due to collinearity with unemployment

### Multivariate analysis

In the multivariate analysis of the EM voting pattern (Table 2), both socio-economic and health variables remained significantly associated with voting patterns. Noticeably, diabetes mellitus was found to be one of the most significant variables (*p* < 0.0001), along with mortality (*p* = 0.0076), and the rate of patients treated for a severe chronic condition as recognized by the French public health insurance (*p* = 0.0021). Asthma only had a trend toward significance (*p* = 0.061). In the multivariate analysis of the MLP voting pattern (Table 3), diabetes mellitus (*p* < 0.0001), COPD (*p* = 0.01), the same significant chronic conditions (*p* = 0.014), mortality (*p* = 0.03), and life expectancy (*p* = 0.032) remained significant along with wealth and social indicators.

### Turnouts and blank votes

**Fig. 4:**
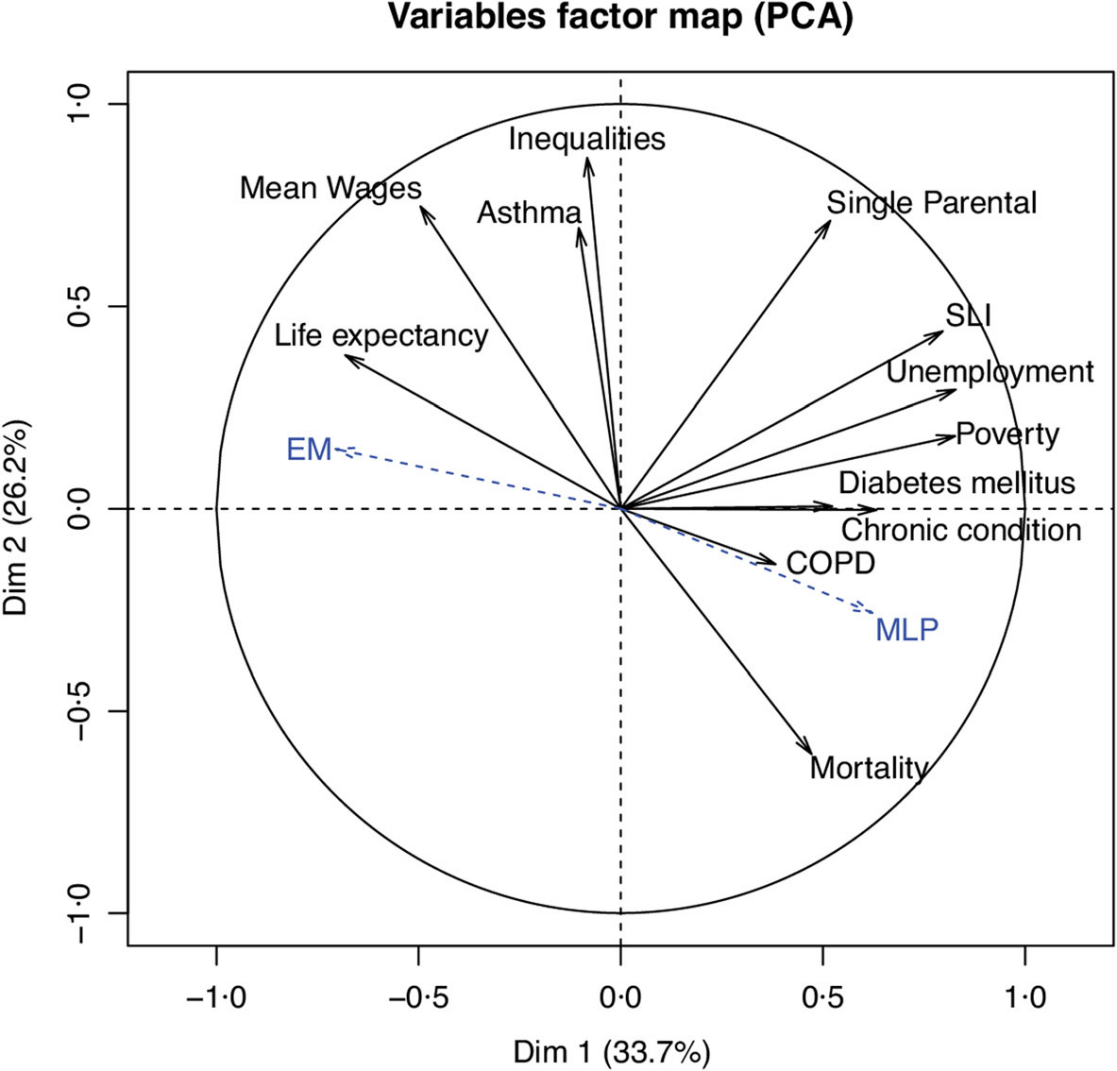
Results of the Principal Component Analysis (PCA). The first factorial plane is presented with DIM1 being the first and DIM 2 the second principal component (with their associated inertia). Axes scales represent correlation with the component/dimension. Variables used for the calculations are then projected onto this plane along with the Emmanuel Macron and Marine Le Pen variables which were not used to construct the axes. EM: Emmanuel Macron, MLP: Marine Le Pen, SLI: Solidarity Labour Income, COPD: Chronic Obstructive Pulmonary Disease

Variables associated with turnouts and blank votes were researched as a sensitivity analysis (Table 4). Solidarity Labour Income, (*p* < 0.001), chronic condition (*p* = 0.008), median annual wage (*p* = 0.037), poverty (*p* < 0.0001), single-parent family (*p* = 0.0002), unemployment (p < 0.0001), life expectancy (*p* = 0.0013), diabetes mellitus (p < 0.0001), sex ratio (*p* = 0.034) and age (*p* = 0.0072) were associated with turn out. Wage (p < 0.0001), single-parent family (p < 0.0001), inequality (p < 0.0001), asthma (p < 0.0001) and age (p = 0.0013) were associated with blank votes. Full results of the sensitivity analysis are available in the Table 4. Moreover, in the principal component analysis, turns out and blanks were projected in directions nearly orthogonal to those of EM and MLP. Notably, turnouts were projected with single parental, SLI, unemployment and poverty (see Fig. 4).

**Table 4:** Variables associated with turnouts and blank votes in the 95 French departments in the univariate analysis

	Turn out			Blank votes		
Variable	Coefficient	Standard error	p-value	Coefficient	Standard error	*p*-value
SLI	119.515	19.265	< 0.0001	−2.441	2.296	0.29
Chronic condition	37.43	10.832	0.00083	0.809	1.158	0.49
Wage	−0.167	0.079	0.037	−0.053	0.006	< 0.0001
Poverty	40.053	6.345	< 0.0001	0.427	0.763	0.58
SPF	29.671	7.73	0.00022	−4.165	0.721	< 0.0001
Inequality	−46.373	52.113	0.38	−38.327	3.507	< 0.0001
Unemployment	64.859	11.896	< 0.0001	0.952	1.375	0.49
Life expectancy	−0.743	0.225	0.0013	−0.103	0.021	< 0.0001
Mortality	− 161.321	123.541	0.19	59.189	11.008	< 0.0001
COPD	86.129	56.956	0.13	3.419	5.81	0.56
Asthma	13.672	10.219	0.18	−4.21	0.947	< 0.0001
Diabetes mellitus	204.214	41.655	< 0.0001	6.156	4.67	0.19
Sex ratio	27.829	12.953	0.034	7.276	1.11	< 0.0001
Age	−0.266	0.097	0.0072	0.032	0.01	0.0013

*SLI*: Solidarity Labour Income; *SPF*: Single-parent family; *COPD*: Chronic Obstructive Pulmonary Disease. Coefficients are coefficients of linear regression. Linear regressions were weighted by department population

### Sensitivity analysis

Sensitivity analysis of linear regression without weighting the regression by population size revealed no significant changes in the results for EM voting pattern. In the MLP voting pattern, two more variables were retained in the final model: asthma (*p* = 0.045) and poverty (*p* = 0.11). In the elastic-net, diabetes mellitus and mortality rates were selected as one of the first and strongest variables, for both EM and MLP. Asthma and the rates of patients with chronic disease for EM and life expectancy for MLP were selected lately with smaller coefficients.

## Discussion

In this analysis of the first round of the 2017 French presidential election, we showed that, even after adjustment on wealth and social confounders, most health-related indicators were strongly associated with voting patterns both for Emmanuel Macron, the “nor right-nor left wing” candidate, and Marine Le Pen, the far-right candidate. Diabetes mellitus, being affected by a severe chronic condition, and mortality were among the strongest predictors in both models. Whereas other researchers have raised the possibility that health recently influenced votes for Brexit and Donald Trump respectively in the United-Kingdom and the US, our findings come to support health as an independent political marker in France also.

Our study has several strengths. First, it is to our knowledge, the first assessment in the medical literature of the relationship between health and voting pattern at a French presidential election. Second, our analysis relies upon official, updated and very reliable data sources. All collected variables came from public registries and databases, which are constructed through rigorous and transparent methods, and revised almost every year. Except for one of them (the Gini coefficient), we retrieved very recent data for each selected indicator. Third, since health is a highly complex and multifaceted concept, we chose an extensive set of relevant indicators, with so-called “hard” endpoints such as mortality and severe chronic conditions. Fourth, because there is a well-established relationship between wealth and health status, [18, 26] we adjusted our analysis on another set of income indicators through sophisticated statistical methods. Fifth, we used a sophisticated and established method for conducting the search of associations between variables and our outcome. Principal component analysis has extensively been used, including in landmark papers [27].

Our findings raise several issues worth considering about the relationship between health and voting behaviour. According to most political analysts, Emmanuel Macron and Marine Le Pen had very different projects with respect to almost all possible aspects. Emmanuel Macron comes from the left wing of the political spectrum, yet he repeatedly claimed from the beginning of his campaign a “nor left-nor right wing” position. His program was liberal, both from an economic and societal standpoint. Conversely and even if she rejects this categorization, Marine Le Pen is considered as supporting a far-right doctrine, expressing among others strong criticisms against liberalism, the European Union project and immigrants. Therefore, it comes with no surprise that social and economic indicators were evenly distributed among both candidates, with departments associated with greater wealth more likely to vote for Emmanuel Macron. However, we also found that most health-related indicators were associated with voting patterns in an independent and very significant way. In our view, this was less expected since France has a healthcare system considered as highly protective and universal [12, 14]. Therefore, and despite significant challenges regarding its sustainability, [12, 14, 28] one might have hypothesized that health would not substantially influence voting behaviour. However, we measured statistical associations that strongly suggest such influence. For instance, we found that when the rate of diabetes mellitus in a given department is around a higher range, people tend to vote less for EM but vote more for MLP. It should be emphasized for non-French readers that the first round of the presidential election did not only include EM and MLP but also 9 other candidates, and that MLP and EM together only attracted 45.31% of all votes. We chose to analyse the results of the first round as it is more likely to reflect the actual voting behaviour *for* a candidate rather than *against* one of them, as it is commonplace at the second round when there are only two options. The ecological nature of our study precludes definitive interpretation of our findings. The most immediate explanation would be that sicker people are more likely to vote for a far-right candidate. Another hypothesis is that people living in areas with deprived health indicators are more likely to choose a candidate perceived as representing a greater potential for change, as compared to the one that already had political responsibilities at the national level. Those assumptions deserve further investigation. Also, further work could search for similar statistical associations between health indicators and results from the second round of the 2017 French presidential election and the first round of the legislative election. Our study also found that socioeconomic factors were related to turnouts and blank votes. However, turnout and blank votes were not associated with the same variables than MLP and EM, suggesting that some factors may influence the decision to vote or not and others may influence the choice of the candidate. This last point was clearly visible after projection on the first factorial plane where MLP and EM projected in the same line but in opposite direction whereas turnout and blank votes combined projected orthogonally to this line.

Our research has limitations, the main one probably being ecological fallacy. Estimated effects are conditional-on-observables and we cannot exclude unobserved confounders. Indeed, this study is based on aggregated data at the department level, not upon individual data. Substantial heterogeneity exists within a department and thus aggregating data may engender a loss of information and statistical power. Additionally, both the socio-economic variables and health variables were calculated on the whole population of the department, but due to French electoral law, not all the population living in a department are voting. There might be a bias as patients who vote may be different from those who do not. Consequently, our findings need to be interpreted with caution. In particular, they do not necessarily mean that people with poorer health had significantly different votes. It can for instance be hypothesized that people unaffected by substantial medical condition but who live in degraded areas were influenced by their environment. Yet, individual patients’ data would have been almost impossible to obtain due to patient doctor privilege and the secrecy of the vote. Instead, high quality aggregates (as measured by French Public Institutes and the Ministry of the Internal Affairs) are reliable and free of the secrecy problem. The French department is the smallest administrative unit for which both voting patterns and indicators were available. Regarding the choice of the statistical model, one can argue that another model (multinomial) or a more complex representation of the relation between the variable could have performed better. We believe that this model was the most adequate choice as it is easier to understand and less prone to over fitting. Moreover, the elastic-net procedures confirmed that the variables we selected in this approach are reliable even in presence of multiple, highly correlated predictors. Last, we selected several health indicators to be incorporated in the model, some of them being reference indicators such as mortality, life expectancy, and being affected by a chronic health condition for instance. Other indicators could have been added to the analysis, namely cancer prevalence, mental health, and cardiovascular diseases, and may deserve future dedicated research. Similarly, other economic variables could have been included in the current analysis, such as economic growth, inflation rate, or even subjective variables.

## Conclusions

Our analysis of the first round of the 2017 French presidential election found strong and independent associations between almost all relevant health indicators and voting behaviours. It emerged that areas where health indicators exhibited worse results were also those which were more likely to favour the far-right candidate, even after adjustment on all wealth and social variables. Conversely, areas with better scores in health variables significantly favoured the more moderate and clearly liberal candidate, who eventually was elected two weeks after. Our findings are consistent with recent reports about Brexit and 2016 US presidential election and highlight the considerable attention that health issues deserve, even in such a protective country like France.

## Data Availability

The datasets used and/or analysed during the current study are available from the corresponding author on reasonable request. Most data were retrieved from the following: the National Institute for Statistics and Economic Studies (INSEE, *Institut National de la Statistique et des Etudes Economiques*, https://www.insee.fr/fr/accueil), the Directorate of Research, Evaluation, and Statistics (DREES, *Direction de la Recherche, des Etudes, des Evaluations et des Statistiques*, https://drees.solidarites-sante.gouv.fr/etudes-et-statistiques/), the French Public Health Insurance (*Assurance Maladie*, https://www.ameli.fr/), the Health Watch Institute (*Institut de Veille Sanitaire*, now merged into another structure called *Santé Publique France*, https://www.santepubliquefrance.fr/), the Observatory of Inequalities (*Observatoire des Inégalités*, https://www.inegalites.fr/), and the Research Institute in Health Economics (IRDES, *Institut de Recherche et Documentation en Economie de la Santé*, https://www.irdes.fr/). All the datasets extracted from the websites of those entities are publicly available. Also, administrative boundaries shapefiles were obtained from the Global Administrative Boundaries project (GADM) from UC Davis (https://gadm.org/index.html). Their data are freely available for academic use. Choropleth map were then generated with the R 3.15 software (The R Core Team, Vienna Austria) and the ggplot2 package (Wickham, 2018, https://ggplot2.tidyverse.org) both open sources software.

## Abbreviations

COPD: Chronic Obstructive Pulmonary Disease
DREES: *Direction de la Recherche, des Etudes, des Evaluations et des Statistiques*
EM: Emmanuel Macron
INSEE: *Institut National de la Statistique et des Etudes Economiques*
IRDES: *Institut de Recherche et Documentation en Economie de la Santé*
MLP: Marine Le Pen
PC: Principal Component
PCA: Principal Component Analysis
SLI: Solidarity Labour Income
UK: United Kingdom
US: United States.
